# Unraveling the Influence of the Preexisting Molecular Order on the Crystallization of Semiconducting Semicrystalline Poly(9,9-di-*n*-octylfluorenyl-2,7-diyl (PFO)

**DOI:** 10.1021/acs.chemmater.2c02917

**Published:** 2022-11-23

**Authors:** Valentina Pirela, Mariano Campoy-Quiles, Alejandro J. Müller, Jaime Martín

**Affiliations:** † POLYMAT and Department of Polymers and Advanced Materials: Physics, Chemistry, and Technology, Faculty of Chemistry, University of the Basque Country UPV/EHU, Paseo Manuel de Lardizabal 3, Donostia-San Sebastián20018, Spain; ‡ Institute of Materials Science of Barcelona, ICMAB-CSIC, Campus UAB, Bellaterra08193, Spain; § IKERBASQUE, Basque Foundation for Science, Plaza Euskadi 5, Bilbao48009, Spain; ∥ Universidade da Coruña, Campus Industrial de Ferrol, CITENI, Esteiro, Ferrol15403, Spain

## Abstract

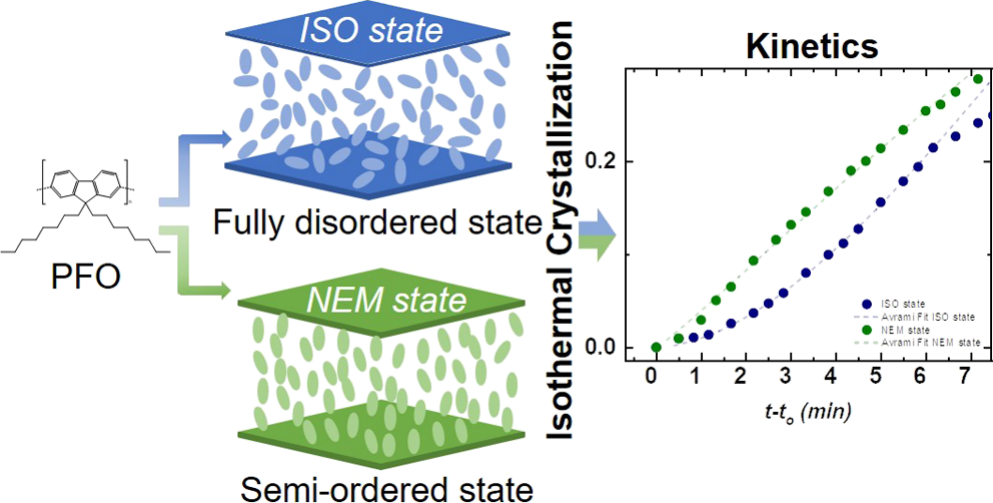

Understanding the complex crystallization process of semiconducting polymers is key for the advance of organic electronic technologies as the optoelectronic properties of these materials are intimately connected to their solid-state microstructure. These polymers often have semirigid backbones and flexible side chains, which results in a strong tendency to organize/order in the liquid state. Therefore, crystallization of these materials frequently occurs from liquid states that exhibit—at least partial—molecular order. However, the impact of the preexisting molecular order on the crystallization process of semiconducting polymers— indeed, of any polymer—remained hitherto unknown. This study uses fast scanning calorimetry (FSC) to probe the crystallization kinetics of poly(9,9-di-*n*-octylfluorenyl-2,7-diyl (PFO) from both an isotropic disordered melt state (*ISO state*) and a liquid-crystalline ordered state (*NEM state*). Our results demonstrate that the preexisting molecular order has a profound impact on the crystallization of PFO. More specifically, it favors the formation of effective crystal nucleation centers, speeding up the crystallization kinetics at the early stages of phase transformation. However, samples crystallized from the *NEM state* require longer times to reach full crystallization (during the secondary crystallization stage) compared to those crystallized from the *ISO state*, likely suggesting that the preexisting molecular order slows down the advance in the latest stages of the crystallization, that is, those governed by molecular diffusion. The fitting of the data with the Avrami model reveals different crystallization mechanisms, which ultimately result in a distinct semicrystalline morphology and photoluminescence properties. Therefore, this work highlights the importance of understanding the interrelationships between processing, structure, and properties of polymer semiconductors and opens the door for performing fundamental investigations via newly developed FSC methodologies of such materials that otherwise are not possible with conventional techniques.

## Introduction

Motivated by the promise of low-cost production of conformable electronic devices, for example, organic solar cells, organic light-emitting diodes, thermoelectric modules, and organic electrochemical transistors, and so forth, semiconducting polymers are attracting great interest from both academic and industrial sectors. The operation of these devices is typically based on the optical and electrical properties of semiconducting polymer thin films, which are known to be intimately connected to their solid-state microstructure. More specifically, properties such as the mobility of charge carriers (either electronic or ionic) and the absorption and emission of light are profoundly affected by the presence of molecular domains with a structural order, for example, crystals, in the material,^1−4^ simply because they exhibit a greater overlap of π-orbitals and a reduced energetic disorder compared to amorphous/disordered domains. Consequently, the control over optical and electrical properties of polymer semiconductors—and hence, rational optimization of devices—stems from a precise understanding and control of their solid-state microstructure.

The solid-state microstructure of many semiconducting polymers is generated via crystallization in the thin film deposition step. Thus, gaining an understanding of how crystallization is developed is of paramount importance to establishing accurate processing-structure–property relationships. However, fundamental investigations of the crystallization process are scarce in the literature and are limited to congeners of the polythiophene family.^5−13^ As a result, many important questions about the crystallization of semiconducting polymers remain unanswered.

Among these, one of the most important questions is how preexisting molecular order in the liquid state impacts crystallization and solid-state microstructure development. Due to the rigidity of aromatic backbones and their amphiphilic nature, many high-performing, crystallizable semiconducting polymers exhibit liquid-crystalline behavior; therefore, crystallization in these polymers likely occurs via the stacking of polymer chain segments that exhibit a preexisting order in the liquid state.^14−17^ Moreover, even nonliquid-crystalline polymer semiconductors are known to exhibit a strong tendency to form aggregates with local molecular order prior to crystallization.^17−21^ Therefore, a major fundamental question in the field remains as to whether or not (and if so, how) the crystallization process of semiconducting polymers is affected by the presence of molecular order in the liquid state.

Crystallizable main-chain liquid-crystalline semiconducting polymers seem ideal material systems to investigate this scientific problem as (i) they can crystallize and (ii) exhibit both ordered and disordered liquid phases. However, because the liquid phase that is thermodynamically stable at temperatures immediately above the crystallization temperature is the liquid-crystalline mesophase, these materials have a strong tendency to crystallize solely from the ordered mesophase. Conversely, the crystallization from the isotropic phase is strongly hampered in these materials. Most likely due to this experimental difficulty, the effect of the preexisting molecular order of an isotropic melt on the crystallization of polymers has been largely unexplored, not just for semiconducting polymers but also for polymers in general.^10,12,22−28^

Fortunately, the above-mentioned experimental difficulties in investigating crystallization from the isotropic phase may be overcome with advanced thermal characterization methods, such as fast scanning calorimetry (FSC). The extremely fast heating and cooling ramps (up to ∼ 10^4^ °C/s) that can be applied in FSC are opening a plethora of new possibilities to investigate materials’ thermal phase transitions, including those previously nonaccessible. For example, potentially, one can design thermal treatments aimed at suppressing liquid-crystalline mesophases (at temperatures slightly above crystallization temperature) so that liquid-crystalline polymers can be crystallized from a disordered, isotropic liquid state.

To explore the hypothesis above, we selected poly(9,9-di-*n*-octylfluorenyl-2,7-diyl (PFO) as a model material system. PFO is a well-known crystallizable semiconducting polymer with relatively low thermal transition temperatures. Therefore, suitable thermal protocols can be designed to minimize the risk of significant degradation issues.^21^ In addition to—at least—two crystalline forms, PFO exhibits a nematic liquid-crystalline mesophase (hereafter referred to as the *NEM state*) in the temperature range immediately above the crystalline phase(s) along with an isotropic liquid phase (hereafter referred to as *ISO state*) at higher temperatures.^11,13,21,22,29−31^

Hence, in this paper, we unravel the impact of the preexisting molecular order on the isothermal crystallization kinetics of the semiconducting polymer PFO, which allows us to rationalize the resulting solid-state microstructure and the optical response (photoluminescence) of the solid material. We discover that the effect of the molecular order on crystallization is complex: the kinetics of the early stages of crystallization is faster when crystallization occurs from the ordered liquid state, likely because the preexisting molecular order facilitates crystal nucleation. However, liquid-crystalline order seems to slow down the advance of the later stages of the crystallization, that is, those governed by molecular diffusion, likely because the chain segments diffusing to the growing crystal front must distort the ordered molecular arrangement in the liquid mesophase, which has an associated free-energy penalty. The different crystallization kinetics result in a distinct dimensionality of the crystallization, which yields different crystal morphologies and, ultimately, a different optical response (photoluminescence) in the semiconducting solid material.

## Experimental Section

### Materials

The PFO sample was donated to us by the group of De Mello and used without further purification.^29^ The polymer had a number-average molecular weight (*M*_n_) of 13.04 kDa, and the dispersity (D̵) was 2.03, as determined by size exclusion chromatography in combination with multiangle light scattering (SEC-MALS) and size exclusion chromatography calibrated with polystyrene (SEC-PS), respectively. THF was purchased from Sigma-Aldrich and used without further purification. PFO thin films were prepared by placing a drop of glucose solution directly on the reference part on the backside of the chip sensor. Then, a 10 mg/mL solution of PFO in THF was deposited by spin coating (2,000 rpm, 60 s) onto the FSC chip sensor. Finally, the glucose drop is removed carefully with water.

### Fast Scanning Chip Calorimetry

Fast scanning chip (FSC) calorimetry experiments were performed on a Mettler-Toledo Flash DSC 2 + device. The equipment is connected to a Huber TC-100 intracooler, permitting scans of up to 40,000 °C/s. The MultiSTAR UFS1 (24 × 24 × 0.6 mm^3^) chip sensors were conditioned and corrected prior to use according to the Flash DSC 2 + specifications. Measurements were carried out under a nitrogen atmosphere, with a constant flow rate of 80 mL/min. The STARe software was used to analyze the data. The thermal protocols essentially consisted in recording heat flow rates during the heating of the sample crystallized at a given temperature at different times. Unless otherwise specified, note that the selected heating and cooling rates for FSC used in this paper were 4000 °C/s. It is important to notice that for FSC measurements, the sample mass is in the nanoscale magnitude. The same sensor was used for all crystallization experiments. Hence, the sample size remained constant.

### Atomic Force Microscopy

A dimension ICON with a Nanoscope V controller (Bruker) atomic force microscopy (AFM) was used to image the samples. A Peak-Force tapping mode using ScanAsyst-Air tips by Bruker (nominal tip radius of 2 nm, nominal frequency of 70 kHz, nominal spring constant = 0.4 N/m) was used to obtain the images. A PFO thin film was deposited from a 10 mg/mL solution on the back side of the chip with a glucose cover on the reference cell, which was removed after deposition with water. The sample was heated above the nematic-to-isotropic transition (*T*_LC-I_), and after melting, it was rapidly cooled (at 4,000 °C/s) to the annealing temperature (*T*_a_). Subsequently, the sample was kept at *T*_a_ for 10 h (the time it reaches maximum saturation), it was rapidly cooled to a temperature below the glassy state, *T*_g_, and rapidly heated to room temperature and then measured by AFM.

### Photoluminescence Spectroscopy

Photoluminescence spectra were measured using Witec equipment. We excited it through a UV high transmission 40× objective using a solid-state laser with a peak wavelength at 355 nm, with a power of 60 μW. We made 500 μm × 500 μm images of the samples directly on the FSC sensor chips. We took a total of 2500 spectra per sample. Cluster analysis of the data revealed three different regions for each chip: (i) the inner part, likely very thick as deduced from the apparent self-absorption features in the spectra; (ii) the region above the heating resistance; (iii) the border between both. The data shown in the article corresponds to the material fraction just over the resistances. A PFO thin film was deposited from a 10 mg/mL solution on the back side of the chip with a glucose cover, after deposition, the drop was eliminated with water. The sample was heated above *T*_LC-I_ to erase the thermal history and then rapidly cooled (at 4,000 °C/s) from the melt to the selected isothermal crystallization temperature. Subsequently, the sample was kept at *T*_a_ for 10 h (the time it reaches maximum saturation), and it was rapidly cooled to a temperature below *T*_g_ and rapidly heated to room temperature.

## Results and Discussion

### Establishment of Suitable Thermal Protocols for the Study

Prior to our investigation, we wanted to scrutinize the possibility of crystallizing PFO from the disordered *ISO state* by cooling the meltdown from a temperature above the nematic-to-isotropic transition (*T*_LC-I_)^29^ to the crystallization temperature, employing cooling rates like those typically applied in regular differential scanning calorimetry (DSC) or polarized-light optical microscopy (PLOM) experiments, namely, <100 °C/min. However, our data (included in Figure 1S of the Supporting Information) unambiguously proved that this range of cooling rates does not suffice to avoid the formation of the liquid-crystalline mesophase during cooling. Hence, in full accordance with our initial premises, conventional DSC and PLOM are not suitable for these studies, and methods enabling faster cooling rates, such as FSC, need to be used instead.

Thus, we started our study by investigating the thermal conditions that allow us to compare crystallizations from the *ISO* and *NEM states*. More specifically, we need first to gain knowledge of the thermotropic phase behavior of PFO, including phase transition temperatures, and, secondly, to figure out the temperature range that allows isothermal crystallization from both the *ISO state* and the *NEM state*.

**Figure 1:**
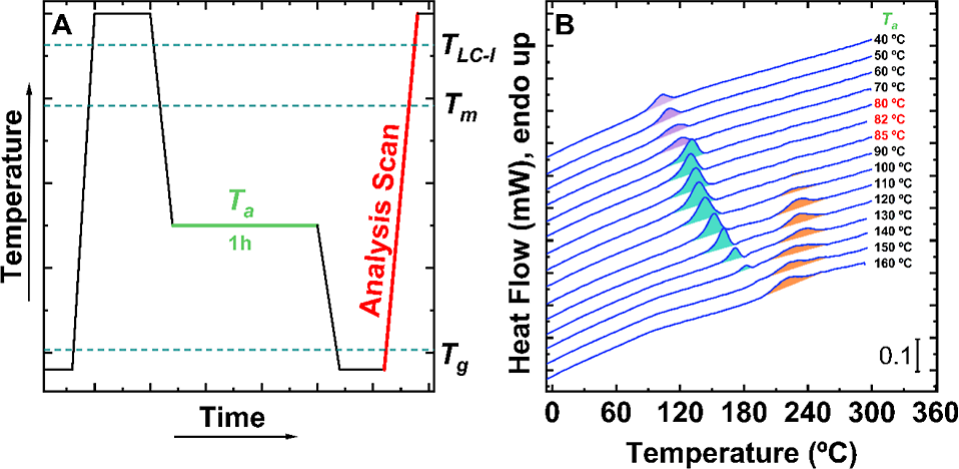
Thermotropic phase behavior of PFO. (A) Thermal protocol employed for the experiment. Relevant temperatures: annealing temperature, *T*_a_, glass transition temperature, *T*_g_, melting temperature, *T*_m_, and nematic-to-isotropic transition temperature, *T*_LC-I_. (B) FSC heating traces (at 4000 °C/s) following the isothermal step of 1 h at temperatures ranging from *T*_a_ = 40 to 160 °C. Endothermic peaks shadowed in purple-cyan and orange correspond to the enthalpic relaxation of the glassy phase, the melting of crystals, and the nematic–isotropic transition, respectively.

Figure 1A shows the FSC thermal protocol used to assess the thermotropic landscape of PFO. First, PFO samples were heated to a temperature well above the *T*_LC-I_ transition to erase any thermal history (e.g., at 300 °C). Then, samples are rapidly cooled down (at 4000 °C/s) to various isothermal temperatures, *T*_a_, ranging from 40 to 160 °C, and kept there for 1 h. During these isothermal steps, the PFO material will evolve according to its thermodynamic nature at that *T*_a_. The evolution suffered by the material at each *T*_a_ is probed in a subsequent heating scan (performed at 4,000 °C/s, identified as an “Analysis scan”).

Figure 1B displays the calorimetric traces corresponding to the heating scans mentioned above (i.e., “Analysis scan”). The *T*_a_ applied in the experiments is indicated on the right-hand side of the curves. Three different endothermic processes can be distinguished in the heating traces depending on the *T*_a_ applied. An aging glass tends to evolve toward an equilibrium state at temperatures well below their *T*_g_. Hence, the area of the physical aging endotherm will decrease as the annealing temperature, *T*_a_, increases, and approaches *T*_g._ Based on this, below *T*_a_ = 80 °C, a broad endotherm at low annealing temperatures (*T*_a_ < 80 °C) is observed. The area of this endotherm decreases with increasing annealing temperature, indicating that PFO is below *T*_g_. That is, the observed endothermic peaks below *T*_a_ = 80 °C correspond to the enthalpic overshoot as a physically aged glass undergoes the glass transition (this peak is shadowed in purple in Figure 1B). Moreover, in cyan color, at temperatures between *T*_a_ = 80 and 150 °C, the overshoot due to the physical aging is no longer visible, and instead, a sharp bell-shaped endotherm associated with the melting process of the crystallites formed during the isothermal steps is observed.^32−34^

Finally, the endothermic peaks colored in orange, from *T*_a_ = 90 °C onward, feature the nematic-to-isotropic transition. Hence, this experiment directly informs about the thermodynamic phase behavior of the material, including relevant transition temperatures.

The experiment above demonstrates that the crystallization of PFO occurs between *T*_a_ = 80 and *T*_a_ = 140 °C. Interestingly, curves obtained for *T*_a_s between 80 °C and below 90 °C feature solely the peak due to the melting of crystals, suggesting that no nematic phase forms during the isothermal steps of 1 h at those temperatures. In other words, the crystallization of PFO at *T*_a_ = 80 °C occurs from the *ISO state* when samples are cooled down from 300 to 80 °C at 4000 °C/s.

**Figure 2:**
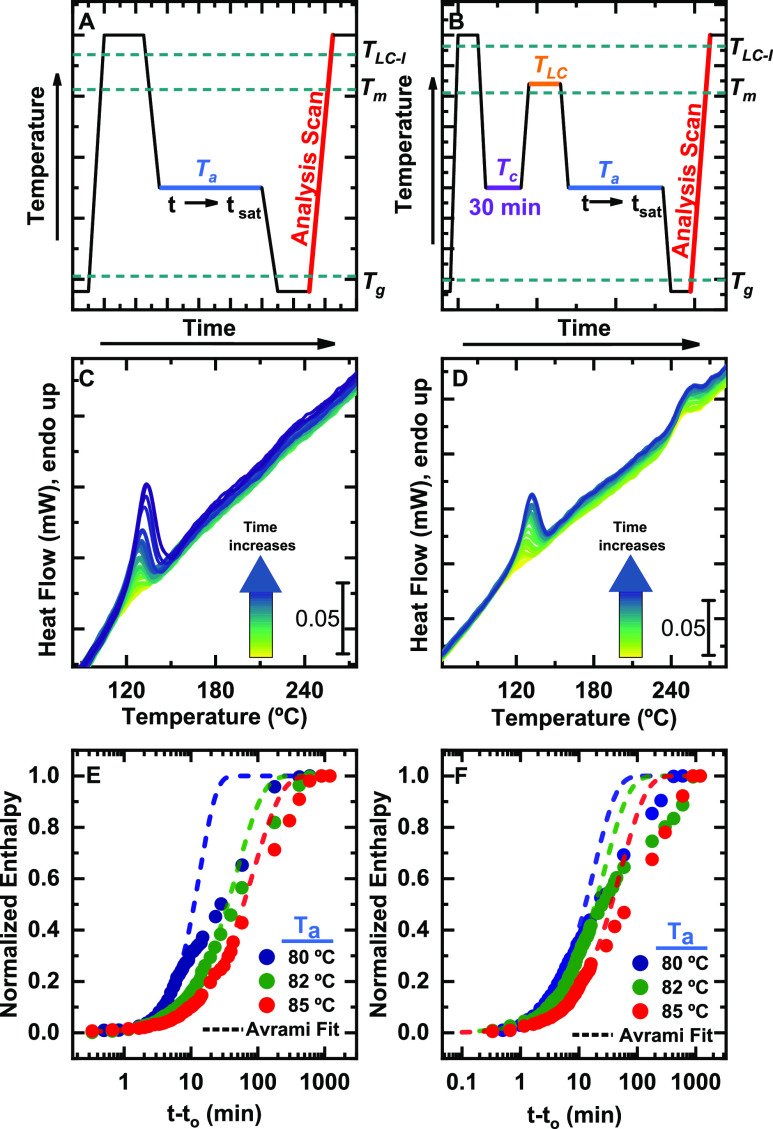
Crystallization from an *ISO state* (left column) and from a *NEM state* (right column) for varying times. (A and B) Thermal protocols used, where *T*_a_ is the annealing temperature, *T*_LC_ is the temperature at which the liquid crystal develops, *T*_m_ is the melting temperature, *t* is the annealing time, and *t*_sat_ is the annealing time when the degree of crystallization reaches saturation. (C and D) FSC heating traces (analysis scans); the progression of time is illustrated by the color scale inside the arrow. (E and F) Advance of crystallization (from normalized enthalpy values) with time at the indicated temperatures and their corresponding Avrami fits. *t* and *t*_*o*_ are the annealing time and the induction time, respectively.

However, an isothermal step of 1 h is typically a short period for the crystallization of polymers to complete at temperatures so close to *T*_g_. Therefore, to identify a suitable temperature range for our study, that is, the temperature range where crystals can develop from the *ISO state*, longer crystallization times need to be explored, for example, 10 h. Shown in Figure 2SA,C and E of the Supporting Information are the calorimetric heating traces after samples were isothermally crystallized for 10 h at *T*_a_s of 80, 82, and 85 °C. As can be observed, neither of those traces exhibits the endothermic peak associated with *T*_LC-I_, proving that PFO crystallizes solely from the *ISO state* between *T*_a_ = 80 and *T*_a_ = 85 °C after being cooled down at 4000 °C/s from 300 °C.

Therefore, a suitable thermal protocol to investigate the crystallization of PFO from the *ISO state* is shown in Figure 2A. The samples are first heated above *T*_LC-I_ to erase the thermal history for a short amount of time of 1 s to avoid degradation. Then, samples are rapidly cooled down (at 4000 °C/s) to the selected *T*_a_ (between 80 and 85 °C), where it is kept for a variable amount of time so that crystallization progresses. Samples are then rapidly cooled down to a temperature below *T*_g_ (at 4000 °C/s), and lastly, they are heated to 300 °C (at 4000 °C/s) for 1 s. The endothermic peak appearing in this heating scan accounts for the melting of crystals formed during the isothermal step; hence, the enthalpy of this melting process can be employed to follow the isothermal crystallization kinetics. It is customary to assume that the values of melting enthalpy (measured under nonisothermal conditions, i.e., during the heating scans after isothermal crystallization) are identical to the values of the crystallization enthalpy developed under isothermal conditions.

Having established the thermal conditions to investigate the isothermal crystallization of PFO from the *ISO state*, we determined a suitable thermal treatment to assess crystallization from the *NEM state* between *T*_a_ = 80 and 85 °C. The *NEM state* can in principle, develop at any temperature above the *T*_m_, but the higher the annealing temperature, the faster the development of the *NEM state*. However, high temperature also prompts undesired thermal degradation processes; hence, we tried to minimize the exposure of samples to high temperatures. We found out that the PFO *NEM state* adequately formed when samples were first crystallized, and then crystals were molten without overpassing *T*_LC-I_. Thus, the thermal protocol to study the crystallization of PFO from the nematic mesophase included two steps (Figure 2B): (i) an initial step in which the mesophase is formed (PFO is crystallized at *T*_a_ = 80 °C for 30 min and then taken to 160 °C for 1 min) and (ii) a second step that is equal to the one employed for the crystallization of samples from the *ISO state*.

## Isothermal Crystallization Kinetics from the Isotropic and the Nematic Liquid States

Once we established the temperature conditions for our crystallization experiments, we endeavored to investigate the overall crystallization kinetics from the disordered *ISO state* and the ordered *NEM state*.

Because polymer crystallization usually proceeds by nucleation and growth, it can be readily modeled with the Avrami framework, which describes the free growth of objects from random nucleation centers. The model yields the expression below (eq 1).^35,36^
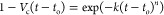
1where *V*_c_ is the relative volumetric transformed fraction to the crystalline state, *t* is the crystallization time, *t*_o_ is the induction time, *k* is the overall crystallization rate constant which includes nucleation and growth components, and *n* is the Avrami index.^35,36^ Moreover, *V*_c_ can be expressed as a function of the mass fraction of the samples (*W*_c_) as seen in eq 2.
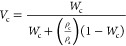
2where *W*_c_ is the mass fraction of the sample, ρ_c_ is the density of a 100% crystalline sample, and ρ_a_ is the density of a 100% amorphous sample. The values of ρ_c_ and ρ_a_ are unknown for PFO, and therefore, we cannot apply eq 1 in terms of volume fraction. However, *V*_c_ is proportional to *W*_c_ (see equations 2 and 3) and the overall crystallization kinetics determined by DSC can be fitted to the Avrami equation in terms of the mass fraction of crystals as an approximation. From eq 3, Δ*H*(*t*) is the enthalpy at a given crystallization temperature, and Δ*H*_total_ is the maximum enthalpy value reached at the end of the isothermal crystallization process. The enthalpies are obtained from the specific heat capacity (*C*_p_) as a function of mass. These values are obtained from the normalized integrated area of each peak divided by the scan rate to determine the relative crystallinity mass fraction of the sample at a given *t* and describe the kinetics of the material at a constant mass.
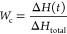
3

Moreover, Müller et al.^37,38^ proposed that the Avrami index (*n*) can be considered in terms of the addition of two components: a nucleation rate component (*n*_n_) and a growth dimensionally (*n*_d_) component (eq 4)*.*^35,36^
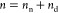
4where *n*_d_ can have values of 1, 2, and 3 depending on the dimensionality of the crystalline ensembles formed [i.e., needles (1D), axialites (2D), and spherulites (3D)]. The *n*_n_ value is proportional to the rate of nucleation with values ranging from 0 to 1; values equal to 1 are due to sporadic nucleation, whereas values equal to 0 represent instantaneous nucleation.

Figure 2C,D shows the heating traces employed for the study of the crystallization kinetics (denoted as “Analysis scan” in Figure 2A,B). For clarity, experimental data obtained at a *T*_a_ of 80 °C is the only one shown here, while data for the rest of the crystallization temperatures are included in the Supporting Information (Figure 2S).

In order to study the crystallization kinetics, the crystal melting peaks were integrated, and the resulting enthalpy (normalized to the final value) was plotted against the crystallization time (Figure 2E,F). We note here too that the heating curves of PFO crystallized from the *ISO state* displayed a single endothermic feature associated with the crystal melting while those of PFO crystallized from the *NEM state* exhibited a further peak associated with *T*_LC-I_ transition.

As *T*_a_ increases, the overall crystallization rate becomes slower. This is reflected in a shift of the curves to higher crystallization times as *T*_a_ increases. In addition, the nucleation rate (1/*t*_*o*_) when crystallizing from an *ISO state* decreases with increasing *T*_a_. *T*_o_ represents the induction time before any crystallization can be detected by the calorimeter; hence, its inverse is proportional to the primary nucleation rate. Therefore, a decrease in 1/*t*_*o*_ indicates that the rate of nucleation becomes slower with increasing *T*_a_. Values of 1/*t*_*o*_ can be found in Table 1S of the Supporting Information.

Interestingly, the comparison of 1/*t*_*o*_ values for crystallizations from the *ISO state* and the *NEM state* at the same temperature reveals that the preexisting molecular order in the crystallizing PFO liquid accelerates the formation of effective nucleation centers. A faster nucleation process can explain why a faster overall crystallization rate is observed in the sample crystallized from the *NEM state* both at 20% conversion (*1/*τ_20%_) and at 50% conversion (*1/*τ_50%_) (see Table 1S of the Supporting Information).

It is important to note that samples crystallized from the *NEM state* require longer times to reach the fully relative crystallized state in the sample, likely suggesting that the liquid-crystalline order slows down the advance of the later stages of the crystallization, that is*,* those governed by molecular diffusion, after the crystallites impinged on one another during the growth process (i.e., during the secondary overall crystallization process that typically occurs at relative conversions to the semicrystalline state larger than 50%).

We should consider that in the process of polymer crystallization, nucleation and mostly free growth from the activated nuclei first take place, and the overall crystallization kinetics accelerates with time (during the so-called primary crystallization). Then, a point is reached at which the kinetics slow down because the growing superstructures [spherulites or axialites, which are semicrystalline entities or lamellar aggregates with three and two dimensions containing amorphous (molten) chains in between them] impinge on one another. This point usually coincides with or is close to 50% conversion to the semicrystalline state and is close to the time to peak when examining isothermal crystallization enthalpy values as a function of time. Secondary crystallization starts at this point when intraspherulitic (or axialitic) and interspherulitic material has not yet crystallized. If we crystallize from a preordered state (i.e., the nematic state), the energy barrier for overall primary crystallization (which includes both nucleation and growth) will most probably be lower than that needed to crystallize from the isotropic melt. We argue that the primary crystallization is dominated by nucleation when it occurs from the nematic state, and the overall crystallization kinetics is accelerated, thanks to the enhanced nucleation with respect to the isotropic state. Then, during secondary crystallization, as amorphous chains are embedded in between the already-formed crystallites, the diffusion rates are usually much lower than during primary crystallization and the effect of nucleation at this stage can almost be neglected.

**Figure 3:**
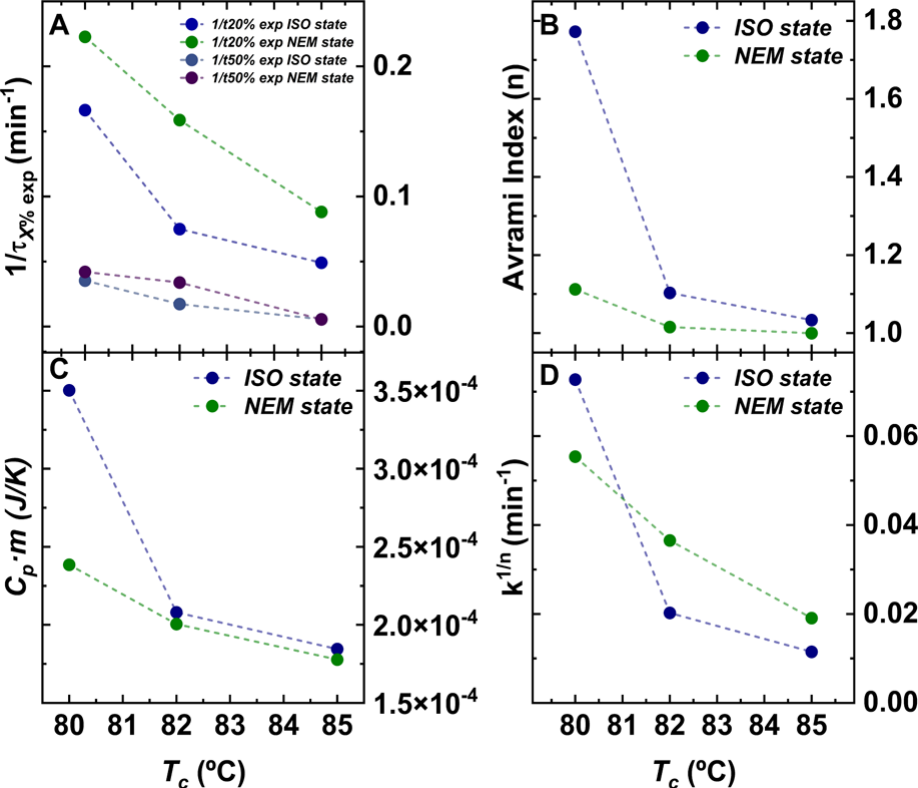
Experimental results and Avrami parameters as a function of crystallization temperature. (A) Experimental values of the inverse of crystallization times (1/τ_50%_ and 1/τ_20%_) for different conversions, (B) Avrami index (*n*), (C) Specific heat capacity for the longest crystallization time times the mass (*Cp·m*) *(*i.e., a proxy for the final degree of crystallinity because *m* is kept constant in the entire experiment), and (D) isothermal crystallization rate obtained from the Avrami model (*k*^*1/n*^).

The *NEM state* exhibits orientational order but lacks the positional order that the crystalline motif has. Therefore, the crystallization of polymer molecules within the *NEM state* must concur with some kind of translational motion that requires molecular or—at least—segmental relaxation. The slower kinetics is thus consistent with the fact that chain segments diffusing to the growing crystal front must distort the ordered molecular arrangement of the *NEM state*, which has an associated free-energy penalty. We must highlight, moreover, that our isothermal crystallization data from in situ wide-angle X-ray scattering (WAXS), included in the Supporting Information (Figure 3S and Table 2S), agree well with the above-mentioned results and conclusions. Clearly, a realistic dynamic microscopic picture—at the molecular level—of how a polymer molecule within the nematic mesophase transits into the crystalline state is required to fully understand the crystallization of PFO polymer.

To gain further information about the crystallization kinetics, experimental data were fitted with the Avrami model (dashed lines in Figure 2E,F). The resulting Avrami parameters are given in Table S1 found in the Supporting Information and plotted in Figure 3. We must note that the Avrami theory is used to describe the primary crystallization range (during the free growth of crystals without any impingement of one another), and fittings for large crystallization conversions are often unsuitable. Hence, to ensure the free growth approximation, the conversion range employed for the fittings was 3–20% of the relative crystallization conversion.

The values obtained for *1*/τ_20%_ by fitting the Avrami theory at 20% conversion are excellent matches to the corresponding experimental values. Hence, as expected, the Avrami theory describes the primary crystallization range very well. However, in the case of 50% conversion, the rate values are overestimated and do not correspond with the experimental values, probably indicating the earlier impingement of crystals in the interval between 20 and 50% conversion, which produces unrealistic fittings at conversions higher than 20%. These deviations of the experimental data from the Avrami fit can be clearly observed in Figure 2E,F.

**Figure 4:**
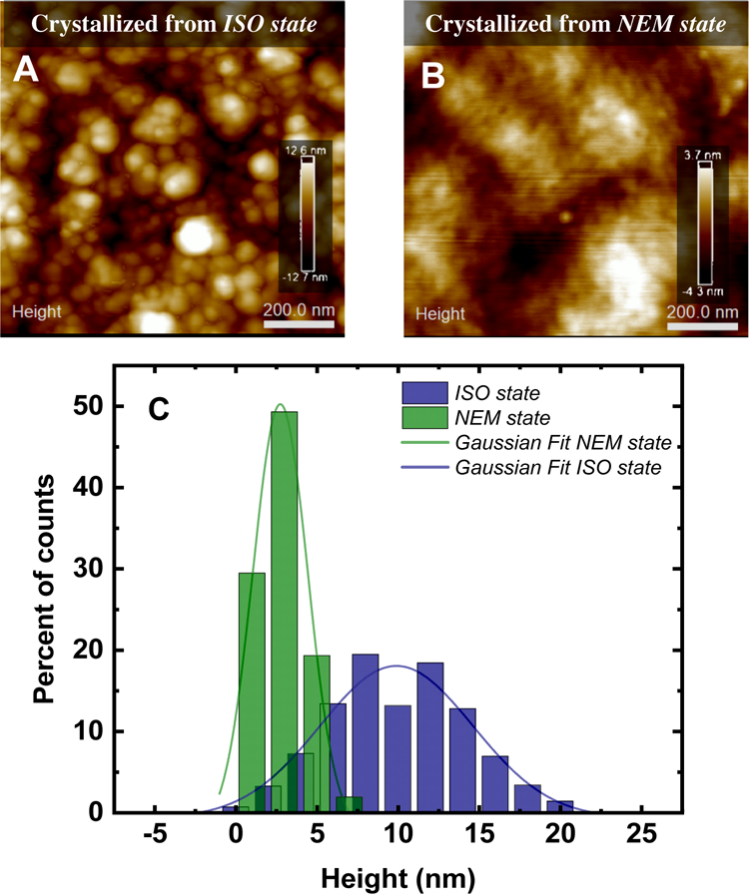
(A) AFM-height images of PFO crystallized at *T*_a_ = 80 °C from an *ISO state*. (B) AFM-height images of PFO crystallized *T*_a_ = 80 °C from a *NEM state*. (C) Height histograms obtained from A and B AFM data. Height distributions in (C) are fitted to Gaussian curves.

Figure 3B shows how the Avrami index (*n*) varies with *T*_a_ values depending on the initial liquid state, that is, *ISO state* versus *NEM state*. The Avrami index was found to be larger at lower *T*_a_ values for crystallization from the *ISO state*. At *T*_a_ = 80 °C, there is a significant change from *n* = 1.7 when the PFO is crystallized from the melt state to 1.1 when it is crystallized from the *NEM state*. That is, the Avrami parameter is closer to 2 for the crystallization from an *ISO state* and to 1 from a *NEM state*. One way to interpret these results is a change in the morphology and nucleation of the PFO. An Avrami index of 2 can be a result of the instantaneous nucleation of axialitic crystals (i.e., two-dimensional aggregates of lamellar crystals), while *n* = 1 could be a result of the instantaneous growth of needle-like crystals. The AFM results (see Figure 4) show some morphological changes that could correspond to the change in the Avrami index. Furthermore, *k*^*1/n*^ is a rate crystallization constant whose values provide information on the overall crystallization rate obtained by the Avrami model which correlate with the obtained experimental values.

Finally, as a proxy for the final degree of crystallinity reached in the samples, the product between the melting enthalpy for the longest crystallization time and the sample mass (*m*) was analyzed, where the sample mass was constant (but unknown) in the whole study (Figure 3C). The results indicate that samples crystallized from an *ISO state* end up being more crystalline than those crystallized from a *NEM state*, especially for the lowest *T*_a_ analyzed. This, again, agrees with our interpretation that chain segment diffusion to the crystal growth front is more impeded in the *NEM state* during the secondary crystallization process.

## Interplay between the Crystallization Kinetics and the Morphology and the Optical Response (AFM and Photoluminescence)

Having established that preexisting molecular order significantly influences the crystallization kinetics of polymers, we analyzed whether the distinct kinetics found result in structural/morphological differences. Thus, samples crystallized both from the *ISO state* and the *NEM state*—thermally treated employing thermal protocols developed for kinetic studies with crystallization times of 10 h—were inspected by AFM (height images).

Tellingly, sample surfaces exhibit markedly different morphology/surface topography. The AFM images and their fast Fourier transform (shown inFigure 4S of the Supporting Information) revealed that PFO crystallized from the *ISO state* develops round-sized nanoscopic features (i.e., axialitic-like), whereas the sample crystallized from the *NEM state* seems to comprise more-elongated features (i.e., needle-like)

The height histograms for the sample crystallized from the *ISO state* exhibit a broad distribution, denoting regions with large height variations, that is, large roughness, whereas histograms for the PFO samples crystallized from the *NEM state* exhibit a narrow distribution of heights, corresponding to a more homogeneous surface (Figure 4C).

Motivated by the aforementioned findings that preexisting order alters the crystallization and the resulting solid-state morphology, we explored whether these changes have in turn an impact on the optoelectronic properties of the semiconducting polymer, for example*,* its optical emission properties (photoluminescence).

**Figure 5:**
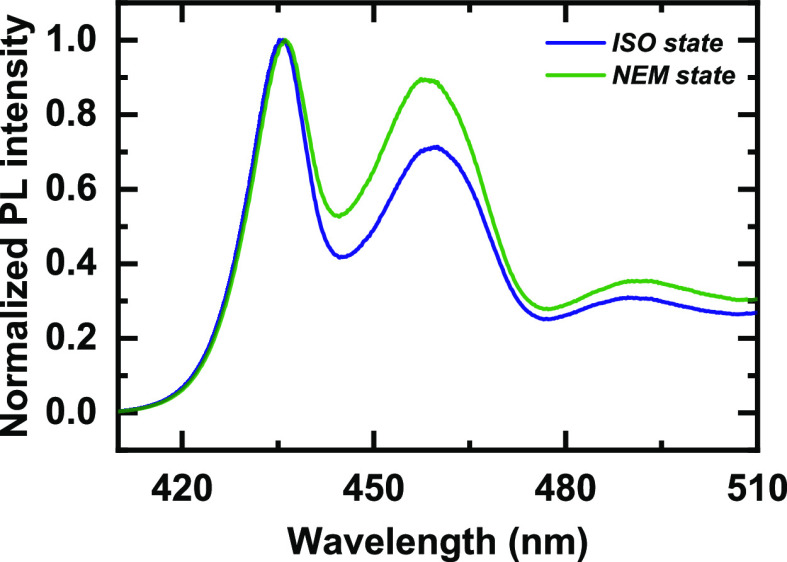
Photoluminescence spectra of crystallized FSC thin-film samples at *T*_a_ = 80 °C from an *ISO state* (blue) and from a *NEM state* (green).

Figure 5 compares the average photoluminescence (PL) spectrum excited at 355 nm for the two samples. The two spectra generally show the same shape: the main 0–0 PL band followed by the first two phonon replicas. The most significant differences are in the relative intensity of the 00 and 01 transitions, with the ratio being smaller for the liquid-crystalline sample and a small blue shift of the PL peaks of the liquid-crystalline sample with respect to the isotropic one. Both of these features suggest a larger fraction of PFO chain segments with planar conformations and hence a larger degree of energetic order for the sample processed from the *ISO state*. This is most likely associated with a higher degree of crystallinity^1^ in this sample as polymers tend to crystallize in lamellar crystals with polymer chains that adopt extended conformations that, in the case of rigid backbone polymers such as PFO, frequently imply planar conformations.

## Conclusions

In this work, we employ fast scanning chip calorimetry to study the crystallization kinetics from different states of a semiconducting semicrystalline material, PFO, opening new possibilities to investigate states of matter that would be otherwise inaccessible through conventional DSC techniques of other polymers in the field of organic electronics. We demonstrate that the preordered molecular domains in the *NEM state* facilitate the formation of effective nucleation sites for crystallization. However, they seem to hinder the diffusion of chain segments to the crystal growth front during the secondary crystallization stage, which slows down the crystal growth process. We argue that the different balance between nucleation and crystal growth between polymers crystallized from ordered and disordered liquids results in distinct solid-state morphologies and different degrees of crystallinity, which eventually impact the optical emission properties of materials. Therefore, our investigation clearly demonstrates a correlation between the preexisting molecular order in the crystallizable liquid, the crystallization kinetics, and the optoelectronic properties of solid semiconducting polymers. However, even more importantly, this work highlights that it is utterly important to conduct more fundamental investigations to gain full control over the optoelectronic properties of organic semiconductors.

## Supporting Information Available

PLOM experiments to determine the microscopic morphology of the material as a function of temperature; melting behavior of isothermal crystallized sample monitored by FSC at different *T*_a_s from both the *ISO state* and *NEM state*; and isothermal crystallization kinetics results from WAXS experiments and FFT analysis of AFM experiments (PDF)

## References

[ref1] Ariu M.; Lidzey D. G.; Lavrentiev M.; Bradley D. D. C.; Jandke M.; Strohriegl P. Study of the Different Structural Phases of the Polymer Poly(9,9′-Dioctyl Fluorene) Using Raman Spectroscopy. Synth. Met. 2001, 116, 217–221. 10.1016/s0379-6779(00)00456-2.

[ref2] Jimison L. H.; Toney M. F.; McCulloch I.; Heeney M.; Salleo A. Charge-Transport Anisotropy Due to Grain Boundaries in Directionally Crystallized Thin Films of Regioregular Poly(3-Hexylthiophene). Adv. Mater. 2009, 21, 1568–1572. 10.1002/adma.200802722.

[ref3] Yang H.; Shin T. J.; Yang L.; Cho K.; Ryu C. Y.; Bao Z. Effect of Mesoscale Crystalline Structure on the Field-Effect Mobility of Regioregular Poly(3-Hexyl Thiophene) in Thin-Film Transistors. Adv. Funct. Mater. 2005, 15, 671–676. 10.1002/adfm.200400297.

[ref4] Noriega R.; Rivnay J.; Vandewal K.; Koch F. P. V.; Stingelin N.; Smith P.; Toney M. F.; Salleo A. A General Relationship between Disorder, Aggregation and Charge Transport in Conjugated Polymers. Nat. Mater. 2013, 12, 1038–1044. 10.1038/nmat3722. 23913173

[ref5] Yu L.; Davidson E.; Sharma A.; Andersson M. R.; Segalman R.; Müller C. Isothermal Crystallization Kinetics and Time-Temperature-Transformation of the Conjugated Polymer: Poly(3-(2′-Ethyl)Hexylthiophene). Chem. Mater. 2017, 29, 5654–5662. 10.1021/acs.chemmater.7b01393. 28713199 PMC5509438

[ref6] Duong D. T.; Ho V.; Shang Z.; Mollinger S.; Mannsfeld S. C. B.; Dacuña J.; Toney M. F.; Segalman R.; Salleo A. Mechanism of Crystallization and Implications for Charge Transport in Poly(3-Ethylhexylthiophene) Thin Films. Adv. Funct. Mater. 2014, 24, 4515–4521. 10.1002/adfm.201304247.

[ref7] Zhao Y.; Yuan G.; Roche P.; Leclerc M. A Calorimetric Study of the Phase Transitions in Poly(3-Hexylthiophene). Polymer 1995, 36, 2211–2214. 10.1016/0032-3861(95)95298-f.

[ref8] Pal S.; Nandi A. K. Cocrystallization Mechanism of Poly(3-Hexyl Thiophenes) with Different Amount of Chain Regioregularity. J. Appl. Polym. Sci. 2006, 101, 3811–3820. 10.1002/app.24067.

[ref9] Yang G. Z.; Chen X.; Wang W.; Wang M.; Liu T.; Li C. Z. Nonisothermal Crystallization and Melting Behavior of a Luminescent Conjugated Polymer, Poly(9,9-Dihexylfluorene-Alt-Co-2,5-Didecyloxy-1,4- Phenylene). J. Polym. Sci. Part B Polym. Phys. 2007, 45, 976–987. 10.1002/polb.21110.

[ref10] Chen X. L.; Huang H. L.; Shi J. G.; Liu Y. L.; Wang L. M. Isothermal Crystallization Kinetics and Melting Behavior of a Luminescent Conjugated Polymer, Poly(9,9-Dihexylfluorene-Alt-2,5-Didodecyloxybenzene). J. Macromol. Sci. Part B Phys. 2012, 51, 1049–1056. 10.1080/00222348.2011.625883.

[ref11] Chen S. H.; Wu Y. H.; Su C. H.; Jeng U.; Hsieh C. C.; Su A. C.; Chen S. A. Cold Crystallization of Poly(9,9-Di-n-Octyl-2,7-Fluorene). Macromolecules 2007, 40, 5353–5359. 10.1021/ma070237g.

[ref12] Yang G. Z.; Chen X.; Xu Y.; Li C. Z.; Wu P.; Liu T. Nonisothermal Crystallization Behavior of a Luminescent Conjugated Polymer, Poly(9,9-Dihexylfluorene-Alt-2,5-Didodecyloxybenzene). Polym. Int. 2007, 56, 245–251. 10.1002/pi.2147.

[ref13] Perevedentsev A.; Stavrinou P. N.; Bradley D. D. C.; Smith P. Solution-Crystallization and Related Phenomena in 9,9-Dialkyl-Fluorene Polymers. I. Crystalline Polymer-Solvent Compound Formation for Poly(9,9-Dioctylfluorene). J. Polym. Sci. Part B Polym. Phys. 2015, 53, 1481–1491. 10.1002/polb.23798. PMC4584509 26435576

[ref14] Bridges C. R.; Ford M. J.; Bazan G. C.; Segalman R. A. Molecular Considerations for Mesophase Interaction and Alignment of Lyotropic Liquid Crystalline Semiconducting Polymers. ACS Macro Lett. 2017, 6, 619–624. 10.1021/acsmacrolett.7b00273. 35650847

[ref15] Zhang L.; Zhao K.; Li H.; Zhang T.; Liu D.; Han Y. Liquid Crystal Ordering on Conjugated Polymers Film Morphology for High Performance. J. Polym. Sci. Part B Polym. Phys. 2019, 57, 1572–1591. 10.1002/polb.24885.

[ref16] McCulloch I.; Heeney M.; Bailey C.; Genevicius K.; MacDonald I.; Shkunov M.; Sparrowe D.; Tierney S.; Wagner R.; Zhang W.; Chabinyc M. L.; Kline R. J.; McGehee M. D.; Toney M. F. Liquid-Crystalline Semiconducting Polymers with High Charge-Carrier Mobility. Nat. Mater. 2006, 5, 328–333. 10.1038/nmat1612. 16547518

[ref17] Bridges C. R.; Ford M. J.; Popere B. C.; Bazan G. C.; Segalman R. A. Formation and Structure of Lyotropic Liquid Crystalline Mesophases in Donor-Acceptor Semiconducting Polymers. Macromolecules 2016, 49, 7220–7229. 10.1021/acs.macromol.6b01650.

[ref18] Marina S.; Gutierrez-Fernandez E.; Gutierrez J.; Gobbi M.; Ramos N.; Solano E.; Rech J.; You W.; Hueso L. E.; Tercjak A.; Ade H.; Martin J. Semi-Paracrystallinity in Semi-Conducting Polymers. Mater. Horizons 2022, 9, 1196–1206. 10.1039/d1mh01349a. 34984421

[ref19] Liu X.; Huettner S.; Rong Z.; Sommer M.; Friend R. H. Solvent Additive Control of Morphology and Crystallization in Semiconducting Polymer Blends. Adv. Mater. 2012, 24, 669–674. 10.1002/adma.201103097. 22109895

[ref20] Liu Y.; Zhao J.; Li Z.; Mu C.; Ma W.; Hu H.; Jiang K.; Lin H.; Ade H.; Yan H. Aggregation and Morphology Control Enables Multiple Cases of High-Efficiency Polymer Solar Cells. Nat. Commun. 2014, 5, 1–8. 10.1038/ncomms6293. PMC4242436 25382026

[ref21] Luzio A.; Nübling F.; Martin J.; Fazzi D.; Selter P.; Gann E.; McNeill C. R.; Brinkmann M.; Hansen M. R.; Stingelin N.; Sommer M.; Caironi M. Microstructural Control Suppresses Thermal Activation of Electron Transport at Room Temperature in Polymer Transistors. Nat. Commun. 2019, 10, 3365 10.1038/s41467-019-11125-9. 31358747 PMC6662673

[ref22] Padmaja S.; Ajita N.; Srinivasulu M.; Girish S. R.; Pisipati V. G. K. M.; Potukuchi D. M. Crystallization Kinetics in Liquid Crystals with Hexagonal Precursor Phases by Calorimetry. Zeitschrift fur Naturforsch. - Sect. A J. Phys. Sci. 2010, 65, 733–744. 10.1515/zna-2010-8-916.

[ref23] Carpaneto L.; Marsano E.; Valenti B.; Zanardi G. Crystallization and Melting Behaviour of a Semirigid Liquid-Crystalline Polyester. Polymer 1992, 33, 3865–3872. 10.1016/0032-3861(92)90374-6.

[ref24] Katerska B.; Exner G.; Perez E.; Krasteva M. N. Cooling Rate Effect on the Phase Transitions in a Polymer Liquid Crystal: DSC and Real-Time MAXS and WAXD Experiments. Eur. Polym. J. 2010, 46, 1623–1632. 10.1016/j.eurpolymj.2010.03.018.

[ref25] Androsch R.; Soccio M.; Lotti N.; Cavallo D.; Schick C. Cold-Crystallization of Poly(Butylene 2,6-Naphthalate) Following Ostwald’s Rule of Stages. Thermochim. Acta 2018, 670, 71–75. 10.1016/j.tca.2018.10.015.

[ref26] Ding Q.; Soccio M.; Lotti N.; Cavallo D.; Androsch R. Melt Crystallization of Poly(Butylene 2,6-Naphthalate). Chinese J. Polym. 2020, 38, 311–322. 10.1007/s10118-020-2354-5.

[ref27] Ding Q.; Jehnichen D.; Göbel M.; Soccio M.; Lotti N.; Cavallo D.; Androsch R. Smectic Liquid Crystal Schlieren Texture in Rapidly Cooled Poly(Butylene Naphthalate). Eur. Polym. J. 2018, 101, 90–95. 10.1016/j.eurpolymj.2018.02.010.

[ref28] Cavallo D.; Mileva D.; Portale G.; Zhang L.; Balzano L.; Alfonso G. C.; Androsch R. Mesophase-Mediated Crystallization of Poly(Butylene-2,6- Naphthalate): An Example of Ostwald’s Rule of Stages. ACS Macro Lett. 2012, 1, 1051–1055. 10.1021/mz300349z. 35607036

[ref29] Martin J.; Davidson E. C.; Greco C.; Xu W.; Bannock J. H.; Agirre A.; de Mello J.; Segalman R. A.; Stingelin N.; Daoulas K. C. Temperature-Dependence of Persistence Length Affects Phenomenological Descriptions of Aligning Interactions in Nematic Semiconducting Polymers. Chem. Mater. 2018, 30, 748–761. 10.1021/acs.chemmater.7b04194.

[ref30] Kawamura T.; Misaki M.; Koshiba Y.; Horie S.; Kinashi K.; Ishida K.; Ueda Y. Crystalline Thin Films of β-Phase Poly(9,9-Dioctylfluorene). Thin Solid Films 2011, 519, 2247–2250. 10.1016/j.tsf.2010.10.048.

[ref31] Elshaikh M.; Marouf A. A. S.; Modwi A.; Ibnaouf K. H. Influence of the Organic Solvents on the α and β Phases of a Conjugated Polymer (PFO). Dig. J. Nanomater. Biostructures 2019, 14, 1069–1077.

[ref32] Wang W.; Fenni S. E.; Ma Z.; Righetti M. C.; Cangialosi D.; Di Lorenzo M. L.; Cavallo D. Glass Transition and Aging of the Rigid Amorphous Fraction in Polymorphic Poly(Butene-1). Polymer 2021, 226, 1–9. 10.1016/j.polymer.2021.123830.

[ref33] Cangialosi D.; Alegría A.; Colmenero J. Cooling Rate Dependent Glass Transition in Thin Polymer Films and in Bulk. In Fast Scanning Calorimetry, 2016, pp 403–431.

[ref34] Martín J.; Stingelin N.; Cangialosi D. Direct Calorimetric Observation of the Rigid Amorphous Fraction in a Semiconducting Polymer. J. Phys. Chem. Lett. 2018, 9, 990–995. 29420893 10.1021/acs.jpclett.7b03110

[ref35] Lorenzo A. T.; Arnal M. L.; Albuerne J.; Müller A. J. DSC Isothermal Polymer Crystallization Kinetics Measurements and the Use of the Avrami Equation to Fit the Data: Guidelines to Avoid Common Problems. Polym. Test. 2007, 26, 222–231. 10.1016/j.polymertesting.2006.10.005.

[ref36] Pérez-Camargo R. A.; Liu G. M.; Wang D. J.; Müller A. J. Experimental and Data Fitting Guidelines for the Determination of Polymer Crystallization Kinetics. Chinese J. Polym. Sci. 2022, 40, 658–691.

[ref37] Müller A. J.; Balsamo V.; Arnal M. L. Nucleation and Crystallization in Diblock and Triblock Copolymers. Adv. Polym. Sci. 2005, 190, 1–63.

[ref38] Balsamo V.; Urdaneta N.; Pérez L.; Carrizales P.; Abetz V.; Müller A. J. Effect of the Polyethylene Confinement and Topology on Its Crystallisation within Semicrystalline ABC Triblock Copolymers. Eur. Polym. J. 2004, 40, 1033–1049. 10.1016/j.eurpolymj.2004.01.009.

