# Supplementary material for: Unraveling the Influence of the Preexisting Molecular Order on the Crystallization of Semiconducting Semicrystalline Poly(9,9-di-n-octylfluorenyl-2,7-diyl (PFO)
Source: Chem Mater. 2022 Nov 23;34(23):10744–51. doi: 10.1021/acs.chemmater.2c02917 (PMC9754006; doi:10.1021/acs.chemmater.2c02917)
Supplement: Supplementary file 1 — cm2c02917_si_001.pdf [file cm2c02917_si_001.pdf]

Supporting information for

Unraveling the influence of preexisting molecular order on the crystallization of semiconducting semicrystalline poly(9,9-di-n-octylfluorenyl-2,7-diyl (PFO)

Valentina Pirela[§], Mariano Campoy-Quiles[†], Alejandro J. Müller[§,‡], Jaime Martín*[§,‡,∥]*

[§] POLYMAT and Department of Polymers and Advanced Materials: Physics, Chemistry, and Technology, Faculty of Chemistry, University of the Basque Country UPV/EHU, Paseo Manuel de Lardizabal 3, 20018, Donostia-San Sebastián, (Spain).

[†] Institute of Materials Science of Barcelona, ICMAB-CSIC, Campus UAB, 08193, Bellaterra, (Spain).

[‡] IKERBASQUE, Basque Foundation for Science, Plaza Euskadi 5, 48009, Bilbao (Spain).

‖ Centro de Investigacións Tecnolóxicas (CIT), Campus Industrial de Ferrol, Universidade da Coruña, Esteiro, 15471, Ferrol, (Spain).

AUTHOR INFORMATION

Corresponding Authors

*Alejandro J. Müller: alejandrojesus.muller@ehu.es

*Jaime Martín: jaime.martin.perez@udc.es

METHODS

Polarized Light Optical Microscope (PLOM)

Polarized Light Optical Microscope (PLOM) experiments were performed by an Olympus BX51 polarized optical microscope using a THMS 600 Linkam hot stage. Experiments were performed using liquid nitrogen for control over temperature and heating and cooling rate. An Olympus SC50 camera linked to the microscope was employed to observe the samples and obtain micrographs.

Wide-Angle X-ray Scattering (WAXS)

Wide-angle X-ray scattering (WAXS) experiments were measured simultaneously at beamline BL11 NCD-SWEET at ALBA Synchrotron Radiation Facility (Barcelona, Spain). FSC sensors were employed to place samples in the beam path. A THMS 600 Linkam hot stage device was employed for temperature control of the samples. WAXS diffractograms were recorded during crystallization of samples from ordered and disordered liquid states. Prior to WAXS analysis. The X-ray energy source amounted to 12.4 eV using a channel cut Si (1 1 1) monochromator (λ = 1.03 Å). The sample-detector distance was 132.6 mm with a 21.2° tilt angle, and chromium(III) oxide was employed to do the calibration (Rayonix LX255-HS detector, Evanston, IL, USA, with a resolution of 1920 × 5760 pixels and pixel size of 44 m^2). A PFO bulk sample was placed on top side of the chip. The sample was heated above the nematic-to-isotropic transition *(T_{LC-I})* to erase the thermal history, then rapidly cooled (at 4,000 °C/s) from the melt to the selected isothermal crystallization temperature, T_a. Subsequently, the sample was kept at T_a for 10h (the time it reaches maximum saturation), it was rapidly cooled to a temperature below T_g, and rapidly heated to room temperature. Samples thus prepared were transported to the WAXS beamline.

RESULTS

Establishment of Suitable Thermal Protocols for the Study

Polarized Light Optical Microscopy (PLOM) experiments were performed to observe the microscopic morphology of the material as a function of temperature. PLOM micrographs were taken at different temperatures during cooling from the melt at 60 ˚C/min. The representative micrographs are shown in

Figure **1**S. The micrograph shown in

Figure **1**SA was taken at a temperature above the clearing point at 300 ˚C, and as expected, a fully isotropic melt (*ISO state*) was observed, as no light can pass through the crossed polarizers. As the sample is cooled, the nematic liquid crystalline state (*NEM state*) can be identified, as seen in

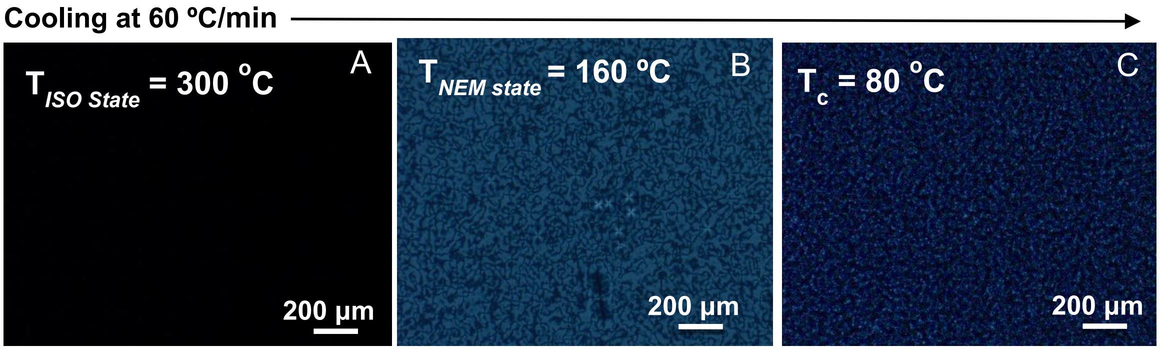

Figure 1S. Polarized light optical micrographs of PFO during cooling at 60 ºC/min. A) 300 ºC, *ISO state.* B) 160 ºC, *NEM state*. C) 80 ºC, crystal phase.

Figure **1**SB, where a weakly birefringent texture can be observed. When the temperature decreases to 80 ˚C, small crystallites in

Figure 1SC were observed, however, their distinction in the micrograph is rather difficult due to their size. This observation of the *NEM state* during cooling indicates that at a cooling rate of 60 ˚C/min, that is, the maximum cooling rate possible when using conventional PLOM and DSC techniques, the study of the kinetics of the material from a completely *ISO state* is not possible as the formation of the *NEM state* during cooling is unavoidable. Therefore, the conventional DSC technique is not suitable for this study and the use of FSC is the technique of choice, as this method enables to cool the sample at significantly faster rates.

Isothermal Crystallization Kinetics from the Isotropic and the Nematic Liquid States

The analysis of the heating curves after the isothermal crystallization for all temperatures are

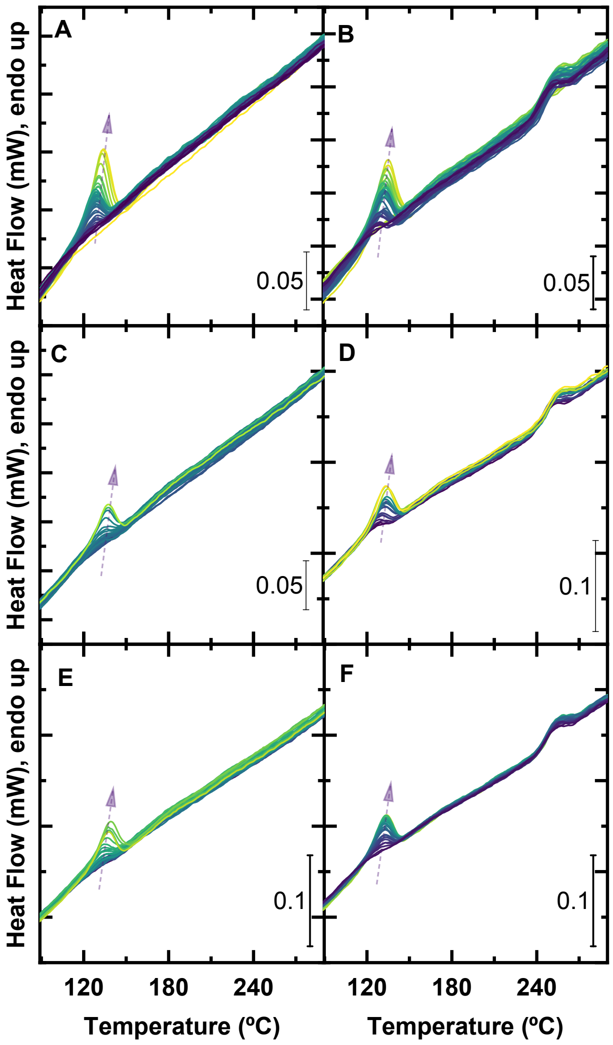

given in Figure 2S.

Figure 2S. FSC heating analysis scans after the isothermal treatment. A) For 80 ºC from *ISO state*. B) For 80 ºC from a *NEM state* C) For 82 ºC from an *ISO state*. D) For 82 ºC from a *NEM state*. E) For 85 ºC from an *ISO state*. F) For 85 ºC from a *NEM state.*

Table S1. Overall crystallization rate parameters (experimental and obtained through the Avrami theory fit)

T_a (°C)	Initial State	$1/t_o$ (min $^{-1}$)	$1/\tau_{20\%}$ exp. (min^{-1})	$1/\tau_{20\%}$ theo. (min^{-1})	$1/\tau_{50\%}$ exp. (min^{-1})	$1/\tau_{50\%}$ theo. (min^{-1})	$k^{1/n}$ (min^{-1})	Avrami index (n)
80	*ISO*	0.630	0.166	0.169	0.035	0.089	0.073	1.7
80	*NEM*	0.857	0.223	0.229	0.042	0.077	0.055	1.1
82	*ISO*	0.480	0.075	0.074	0.017	0.028	0.020	1.1
82	*NEM*	2.00	0.159	0.160	0.034	0.052	0.036	1.0
85	*ISO*	0.46	0.049	0.049	0.0056	0.016	0.011	1.0
85	*NEM*	2.22	0.088	0.089	0.0055	0.027	0.019	0.99

Isothermal Crystallization Kinetics from X-ray Scattering

In these experiments, the sensor with a previous thermal treatment done in the Flash DSC was placed perpendicular to the incident beam and in order to properly measure the sample and ensure a high S/N ratio, it was necessary to deposit the sample on the FSC sensor in the bulk, contrary to

the FSC experiments which were measured in thin-films. The kinetics were followed by measuring the relative crystallinity from the peak increase at q = 15 nm^{-1}, indexed to the crystalline plane [530], with increasing time (Figure 3S). The reflections found on the crystallized sample were able to be indexed to the orthorhombic unit cell of the α-crystal phase of PFO for both samples.[1] Results seen in Figure 3S reveal that for both methods (*i.e.,* FSC and WAXS) similar experimental results are obtained for both crystallization protocols. In addition, after applying the Avrami theory, overall crystallization kinetics ($k^{1/n}$) were significantly different for the two techniques. This could be simply explained since the analysis of the kinetics is done by following a single peak, that is, only one direction of the crystal growth has been analyzed. or due to different sample shape, that is in the bulk compared to thin-film. This signal is in the region of π-π stacking (010) so it is possible the stacking is perpendicular to the growth of the crystal and is inhibited in this direction and growing in another, which is not able to be observed in that direction. However, it was found that the Avrami index gives the same pattern for each initial crystallization state. That is, an Avrami index of 2 for an initial *ISO state* and 1 for an initial *NEM state.*

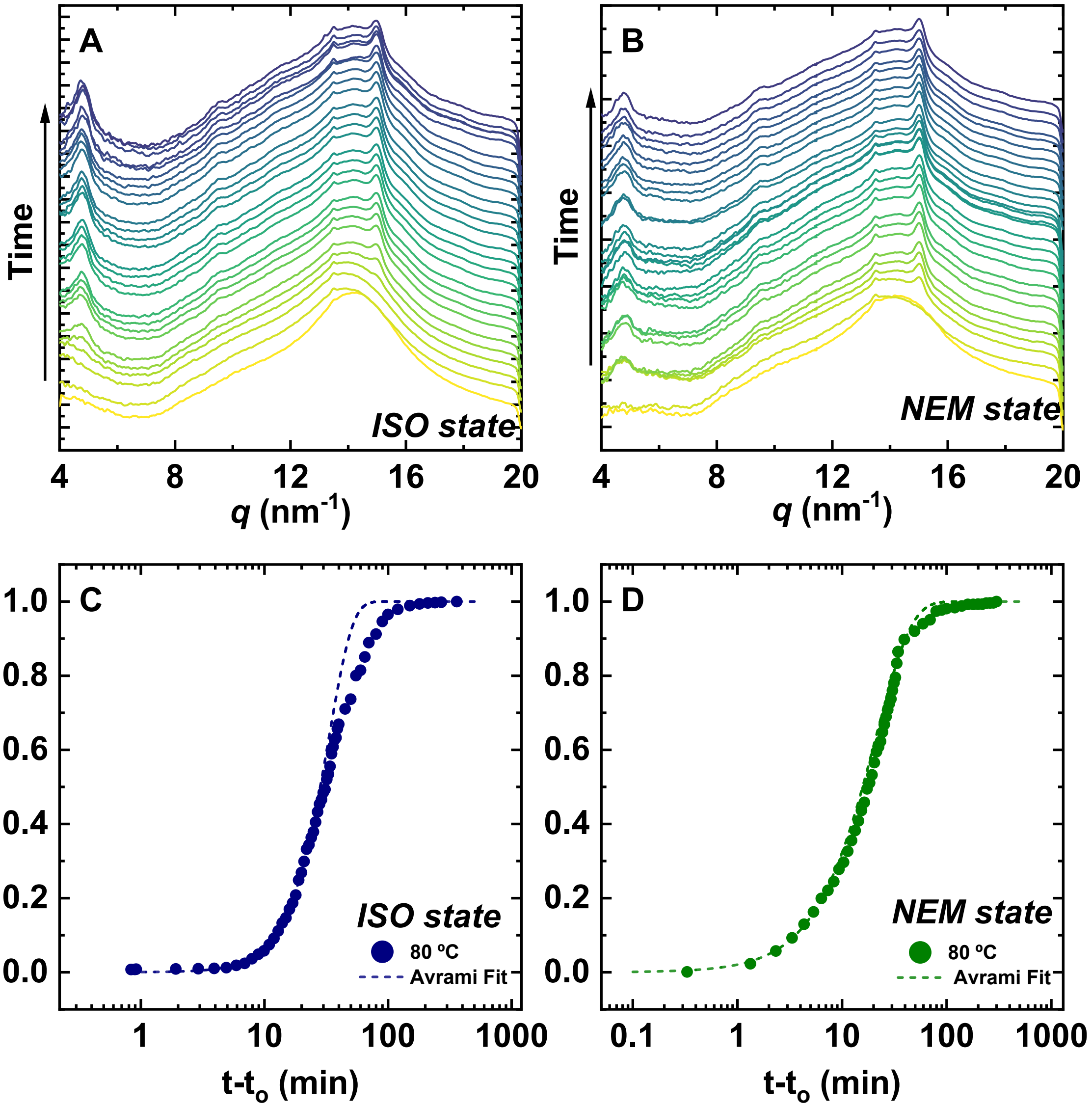

Figure 3S. A and B) WAXS profiles after isothermal treatment for varying times at 80 °C for *ISO state* and *NEM state* crystallization, respectively. C and D) WAXS profiles after isothermal treatment and their corresponding Avrami fittings for an *ISO state* and for a *NEM state*, respectively.

In Table 2S, comparisons between the two techniques are presented to outline comparabilities and differences in the results.

Table 2S. WAXS and FSC Avrami Parameters at $T_a = 80$ °C.

**Technique**	**Initial State**	***1/τ$_{20\%}$* *exp.* (min^{-1})**	***1/τ$_{20\%}$* *theo.* (min^{-1})**	***1/τ$_{50\%}$* *exp.* (min^{-1})**	***1/τ$_{50\%}$* *theo.* (min^{-1})**	**$k^{1/n}$ (min^{-1})**	**Avrami index (n)**
FSC	*ISO*	0.166	0.167	0.035	0.089	0.073	1.7
WAXS	*ISO*	0.056	0.055	0.032	0.035	0.050	2.3
FSC	*NEM*	0.222	0.224	0.042	0.077	0.055	1.1
WAXS	*NEM*	0.159	0.158	0.053	0.061	0.12	1.3

Fast Fourier Transform (FFT)

In FFT images of the phase of the AFM image, we are able to see a structure independently of its height. In the case of the image from *ISO state*, its FFT shows a vertically oriented ellipsoid. This shape is associated with globular domains in the real space with mean axis of around 30 nm (vertical) and 50 nm (horizontal). In the case of the *NEM state*, the FFT image represents elongated

domains with mean axis of 25 nm and 50 nm with two defined lobules associated with a correlation length of 50-60 nm, which is the double of the short axis. That means that the morphology of the *NEM state* is composed of correlated stackings of fibrillar domains, unlike the *ISO state*, in which the array of elongated domains do not show any correlation.

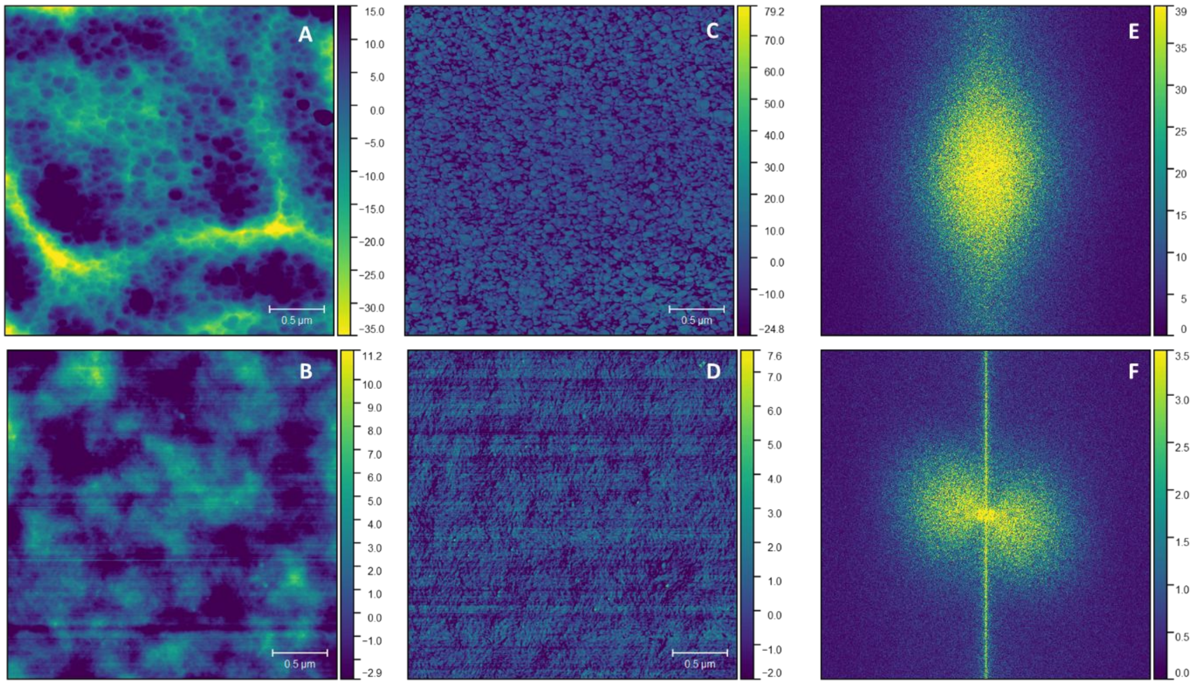

Figure 4S. AFM images. A and B) Height images from ISO state (A) and from NEM state (B). Phase images from ISO state (C) and from NEM state (D). F and G) FFT images from ISO state (F) and from NEM state (G).

REFERENCES

(1) Chen, S. H.; Chou, H. L.; Su, A. C.; Chen, S. A. Molecular Packing in Crystalline Poly(9,9-

Di-n-Octyl-2,7-Fluorene). *Macromolecules* **2004**, *37* (18), 6833–6838.
